# Gene-Diet Interactions in Type 2 Diabetes: The Chicken and Egg Debate

**DOI:** 10.3390/ijms18061188

**Published:** 2017-06-02

**Authors:** Ángeles Ortega, Genoveva Berná, Anabel Rojas, Franz Martín, Bernat Soria

**Affiliations:** 1 Centro Andaluz de Biología Molecular y Medicina Regenerativa–CABIMER, 41092 Seville, Spain; maortega@upo.es (A.O.); gberamo@upo.es (G.B.); anabel.rojas@cabimer.es (A.R.); 2 Universidad Pablo Olavide, 41013 Seville, Spain; 3 Centro de Investigación Biomédica en Red de Diabetes y Enfermedades Metabólicas Asociadas (CIBERDEM), 28029 Madrid, Spain

**Keywords:** Type 2 diabetes, nutrients, nutrigenetic, nutrigenomic, epigenetic, pancreatic β-cell

## Abstract

Consistent evidence from both experimental and human studies indicates that Type 2 diabetes mellitus (T2DM) is a complex disease resulting from the interaction of genetic, epigenetic, environmental, and lifestyle factors. Nutrients and dietary patterns are important environmental factors to consider in the prevention, development and treatment of this disease. Nutritional genomics focuses on the interaction between bioactive food components and the genome and includes studies of nutrigenetics, nutrigenomics and epigenetic modifications caused by nutrients. There is evidence supporting the existence of nutrient-gene and T2DM interactions coming from animal studies and family-based intervention studies. Moreover, many case-control, cohort, cross-sectional cohort studies and clinical trials have identified relationships between individual genetic load, diet and T2DM. Some of these studies were on a large scale. In addition, studies with animal models and human observational studies, in different countries over periods of time, support a causative relationship between adverse nutritional conditions during in utero development, persistent epigenetic changes and T2DM. This review provides comprehensive information on the current state of nutrient-gene interactions and their role in T2DM pathogenesis, the relationship between individual genetic load and diet, and the importance of epigenetic factors in influencing gene expression and defining the individual risk of T2DM.

## 1. Introduction

Diabetes is considered to be one of the biggest global health burdens of the current century. The International Diabetes Federation (IDF) Atlas 2015 [1] estimates that 415 million adults suffer from this disease. In addition, diabetes prevalence is constantly increasing. There are three main types of diabetes (Type 1, Type 2 and gestational diabetes). Type 2 diabetes (T2DM) is the most common type of diabetes. According to IDF, in high-income countries, up to 91% of adults with diabetes have the Type 2 form. The disease usually appears in adults, mostly due to an inappropriate lifestyle, however, T2DM is increasing in children and adolescents.

T2DM is a very complex disease, for which the hallmarks are β-cell failure and insulin resistance (IR). A combination of genetic, epigenetic, environmental, and lifestyle factors, such as diet, are responsible for the onset and development of T2DM [2]. Genome-wide association studies have identified many genetic variant loci involved in T2DM, however, these variants can only explain between approximately 10–15% of the heritability of the disease [3]. Strong evidence in the field proposes epigenetic factors as regulators of gene expression in T2DM. Epigenetic marks might be influenced by environmental factors that ultimately have an impact on the risk of T2DM [4].

The nutrient-gene interactions, as well as the relationship between individual genetic load and diet play an important role in the pathophysiology of T2DM [5]. Thus, a better knowledge of nutrigenomics and nutrigenetics of T2DM, together with a better understanding of the epigenetic marks are instrumental in the development of precision medicine approaches to prevent, detect and treat this disease.

In this review, we will examine the nutrient-gene interactions and their role in T2DM pathogenesis. We will discuss how an individual’s genetic component can also impact on the risk of T2DM, depending on the type of diet they follow. Finally, since epigenetic variations may interact through diet to influence gene expression and define the individual risk of T2DM, we will illustrate the importance of epigenetic factors in explaining the missing heritability cause of T2DM.

## 2. Effects of Nutrients on Gene Expression in the Pathogenesis of T2DM

Dietary nutrition plays an important role in the pathogenesis of T2DM, making the identification and analysis of nutrient-gene interactions a necessary step in the understanding of this chronic disease. Nutrigenomics, the area of nutrition that uses molecular tools to elucidate the influence of nutrients over the genome, proteome and metabolome, provides a genetic understanding of how common dietary components lead to up and/or down-regulation of gene(s) and changes in protein expression levels [6]. The nutrient gene interaction may modulate the gene expression via different mechanisms: (i) Directly; (ii) Through their metabolites; (iii) By activating various signalling molecules of complex metabolic pathways [7].

### 2.1. Polyphenol-Gene Interactions in T2DM Pathogenesis

Polyphenols are a large and heterogeneous group of plant products found in fruits, vegetables, legumes, cereals and chocolate, which may be further classified into flavonoids, lignans, phenolic acids, and stilbenes [8]. Numerous studies have reported beneficial effects of polyphenol-rich foods in lowering the risk of T2DM and improving inflammation and glycaemia markers in Type 2 diabetic subjects [9,10]. Dietary polyphenolic compounds may exert hypoglycemic effects in multiple ways, such as diminished carbohydrate digestion and glucose absorption, inhibition of glucose release, stimulation of insulin secretion and protection of pancreatic β-cells against glucotoxicity, increased glucose uptake in peripheral tissues by modulating intracellular signalling, antioxidant activity and inhibition of advanced glycation end product formation [9,11,12].

#### 2.1.1. Flavonoid-Gene Interactions in DM Pathogenesis

Dietary flavonoids include flavones, flavonols, flavanols, flavanones, isoflavones, and anthocyanins. Numerous studies strongly support the protective effects of polyphenols on glucose homeostasis, and on some specific molecules such as flavanols, quercetin, luteolin and others that have been shown to impact on different steps of intracellular signalling pathways (insulin secretion, insulin signalling and glucose uptake, enhancing mitochondrial status, suppression of inflammatory cytokine production and reactive oxygen species (ROS)/reactive nitrogen). In addition, flavonoids, in a similar way to phenolic acids and tannins, have the property of inhibiting the α-glucosidase and α-amylase enzymes responsible for carbohydrate digestion [9,11,12,13,14,15].

Consistently, human urinary excretion studies on T2DM case-controlled pairs selected from the Nurses’ Health Study (NHS) and NHSII have indicated that specific flavonoids, including flavanones (naringenin and hesperetin) and flavonols (quercetin and isorhamnetin), as well as the phenolic acid, caffeic acid, but not flavan-3-ols and ferulic acid, were associated with a lower risk of developing T2DM in a time-dependent manner [16]. In an average 8.8 year follow-up study in women after ingestion of total or individual flavonoids and flavonoid-rich foods it was shown that whereas total flavonols and flavones, quercetin, kaempferol, myricetin, apigenin, and luteolin did not significantly increase diabetes risk, apple or tea consumption was inversely associated with T2DM risk [17]. This is in accordance with the Health Professionals Follow-Up Study which also found that higher consumption of anthocyanins, particularly from pears, apples and blueberries, but not total flavonoid intake or other flavonoid subclasses, were inversely associated with T2DM [18]. By contrast, the Iowa Women’s Health prospective cohort study in postmenopausal women suggested that only food constituent flavonoid consumption was not associated with diabetes risk. However, in the same study, regular consumption of red wine, white wine, beer, and liquors was inversely associated with diabetes, suggesting that it is the alcohol per se and not the polyphenols which may protect against diabetes [19]. The conflicting results of those studies might be a consequence of the difficulties in evaluating the contribution of other dietary constituents, or improper timing and quality of dietary assessment.

Flavan-3-ols are present in fruit, tea, cocoa and chocolate (Table 1). Catechins are flavanols compounds present mainly in green tea. In streptozotocin (STZ)-induced diabetic rats, catechin hydrate treatment reduced the elevated level of serum glucose and prevented vascular endothelial dysfunction through the activation of endothelial phosphatidylinositol 3-kinase (PI3K) signal and subsequent activation of eNOS signalling systems [20].

One of the most common flavanols is epigallocatechin gallate (EGCG). In rats, EGCG has been demonstrated to affect numerous processes, including more efficient insulin secretory function, increased viability of β-cells under conditions of glucotoxicity, glucose uptake and tolerance, IR, oxidative stress, inflammation and mitochondrial function. These effects of EGCG involved augmented insulin receptor substrate-2 (IRS2) signalling, although other mechanisms might be relevant too. The reductive glucolipotoxic effect of EGCG occurs through the incrementation of AMP-activated protein kinase (AMPK) signalling to inhibit the activities of lipogenic enzymes and ameliorate mitochondrial function [21]. Similarly, the positive effects of EGCG on IR in dexamethasone-induced rat L6 cells was essentially dependent on the AMPK and PI3K/Akt activation pathway [22], while in differentiated rat L6 myotubes, EGCG and epicatechin enhanced insulin-mediated glucose uptake in vitro and translocation of glucose transporter (GLUT) 4 [23]. EGCG has also been shown to protect insulin-producing β-cells from pro-inflammatory cytokine-induced cytotoxicity via the modulation of B cell CLL/lymphoma 2 (BCL-2) expression [24].

In animal studies, EGCG supplementation (1% in diet) in obese db/db mice improved glucose tolerance, increased glucose-stimulated insulin secretion from pancreatic β-cells and preserved islet morphology by reducing the expression of the endoplasmic reticulum stress-associated islet marker genes *DNA-damage inducible transcript 3* (*Ddit3*) and its downstream target protein *phosphatase 1*, *regulatory subunit 15A* (*Ppp1r15a*), as well as *cyclin-dependent kinase inhibitor 1A* (*Cdkn1a*) [25]. In rodent models of T2DM, EGCG enhanced oral glucose tolerance in severely diabetic mice and in moderately diabetic rats, and increased glucose-stimulated insulin secretion. EGCG upregulated glucokinase, acyl CoA oxidase-1 (ACO-1) and carnitine palmitoyl transferase-1 (CPT-1) mRNA expression in liver and adipose tissues of obese db/db mice in a dose-dependent manner. Conversely, exposure of H4IIE hepatoma cells to EGCG decreased the expression of genes involved in gluconeogenesis and the synthesis of fatty acids, triacylglycerol and cholesterol, whereas genes involved in glucose transport (*Glut 1*) and glycolysis (phosphofructokinase) were induced in [26].

In humans, results from epidemiological studies on EGCG are contradictory. Whereas some studies have demonstrated that an acute, high dose of EGCG concentrated green tea supplement could control postprandial hyperglycemia [27], long-term studies in Type 2 diabetic adults did not reveal a hypoglycemic effect [28]. A recent meta-analysis of clinical human studies with catechins in controlled trials on glycemic control showed that green tea catechins (EGCG) reduced fasting glucose over a median of 12 weeks, whereas fasting insulin, glycated haemoglobin (HbA1c), and homeostatic model assessment of IR (HOMA-IR) were not affected [29].

Naringin and hesperidin, the two major flavanones, are present in citrus fruits and also have hypoglycemic and hypolipidemic action in T2DM by partly regulating the fatty acid and cholesterol metabolism and affecting the gene expression of glucose-regulating enzymes (Table 1). In C57BL/KsJ-db/db mice, both hesperidin and naringin significantly increased hepatic glucokinase mRNA level via peroxisome proliferator-activated receptor gamma (PPAR-γ) and upregulated adipocyte *Glut 4*. Naringin also lowered hepatic mRNA expression of phosphoenolpyruvate carboxykinase PEPCK and glucose-6-phosphatase G6Pase [30].

T2DM rats fed a high-fat diet (HFD) and administered low-dose STZ injection naringin, were shown to improve β-cell function, ameliorating hyperglycemia, hyperinsulinemia and IR by increasing PPAR-γ and heat shock proteins (HSP) expression in the livers of diabetic rats [31]. Despite this promising data, more clinical studies in humans to support the anti-diabetic potential of these flavanones are necessary.

The most abundant flavonoids are flavonols, which are widely distributed throughout plant-based foods. Quercetin is one of the most important dietary flavonols and is found in red wine and numerous fruits, vegetables and nuts (Table 1). The Protective effects of quercetin have been related to decreasing oxidative stress with preservation of pancreatic β-cell integrity in STZ-induced diabetic rats, possibly by decreasing lipid peroxidation, nitric oxide (NO) production and by increasing antioxidant enzyme activity [32]. In addition, quercetin seems to be beneficial for the treatment of diabetic neuropathy as it has been shown to protect cultured dorsal root ganglion neurons of rats against high glucose-induced apoptosis via Nrf-2/HO-1 activation and nuclear factor kappa B (NF-κB) inhibition [33]. The inhibitory effect on inflammatory pathways (activation of NF-κB signalling and reduction in serum level of both TNF-α and C-reactive protein (CRP)) without affecting glucose levels, would explain the potential of quercetin to prevent diabetic vascular complications in both animal models with insulin deficiency and resistance by its anti-inflammatory effect rather than its metabolic effects [34]. In a different study, dietary supplementation with 0.5% quercetin in the diet for 2 weeks in STZ-induced T2DM BALB/c mice decreased blood glucose and enhanced serum insulin concentrations by inhibiting expression of *cyclin-dependent kinase inhibitor p21* (*WAF1/Cip1*) (*Cdkn1a*) associated with cell proliferation in the liver and pancreas [35].

Quercetin also showed anti-inflammatory effects in male C57Bl/6j mice and obese Zucker rats. In male C57Bl/6j mice, supplementation with 0.8% quercetin in a HFD for 8 weeks decreased the circulating markers of inflammation interferon-gamma (IFN-γ), interleukin-1alpha (IL-1α), and interleukin-4 (IL-4) [36]. In obese Zucker rats, administration of both 2 and 10 mg/kg of body weight of quercetin for 10 weeks improved dyslipidemia, hypertension, and hyperinsulinemia, but only the highest dose of quercetin produced anti-inflammatory effects as it increased the plasma concentration of adiponectin, reduced NO*_x_* levels in plasma and lowered TNF-α production in visceral adipose tissue [37].

The hypoglycemic effects of the major dietary flavones apigenin and luteolin, found in celery, parsley and many herbs, may be related to the protection of pancreatic β-cells against glucotoxicity (Table 1). In addition, together with quercetin, apigenin, and luteolin protected RINmF5 cells from cytokine-induced pancreatic-cell damage by inhibiting inducible nitric oxide synthase (iNos) gene expression through suppression of NF-κB activation, and also prevented IL-1β- and IFN-γ-mediated inhibition of insulin secretion [38]. However, it is not clear whether this effect also occurs in the islets in vivo.

The major dietary isoflavones are daidzein and genistein which are primarily present in soybeans, soy foods and legumes, and have been shown to reduce hyperinsulinemia (Table 1). Studies in rats with diet-induced obesity showed that soy isoflavones in a HFD significantly stimulated insulin secretion, decreased PPAR-γ, GLUT 2, and SREBP-1 expression, and ameliorating hyperinsulinemia observed during obesity [39]. In STZ-induced diabetic mice, dietary intake of genistein significantly improved hyperglycemia, glucose tolerance, and circulating insulin levels, preserving islet β-cell proliferation, survival, and mass. The insulin-secreting activity and proliferative effect of genistein in pancreatic β-cells is mediated, at least partially, via activation of the cAMP/PKA-dependent ERK1/2 signalling pathway [40]. 

In humans, the antidiabetic effect of the isoflavones is still poorly known. However, data from a human study showed that genistein administration at 54 mg/day in postmenopausal women decreased fasting glucose and increased glucose tolerance and insulin sensitivity [41].

Previous studies have shown that anthocyanins and anthocyanidins stimulate insulin secretion and have protective effects on β-cells in vitro (Table 1) by stimulating important factors for insulin gene transcription and reducing reactive oxygen species (ROS)-mediated apoptosis and necrosis [42]. Intake of anthocyanins in T2DM mouse models was found to inhibit elevation of blood glucose levels and improve insulin sensitivity. The antidiabetic effects of anthocyanins were likely mediated by upregulation of *solute carrier family 2 members 4* (*Slc2a4*) (gene encoding for GLUT 4 transporter), down-regulation of retinol-binding protein and the related inflammatory adipocytokines [43] Similar results were obtained when T2DM mice received a dietary bilberry extract rich in anthocyanins. These mice displayed AMPK activation and *Slc2a4* in white adipose tissue while in liver glucose production and lipid content were suppressed. Inactivation of acetyl-CoA carboxylase and upregulation of PPAR-α, acyl-CoA oxidase, and carnitine palmitoyltransferase-1A were also observed in the liver of TD2M mice fed with anthocyanins [44]. In humans, consumption of anthocyanin-rich foods, particularly blueberries and apples or pears, was associated with a lower risk of T2DM, without a significant association between total flavonoid intake or other types of flavonoids [18].

#### 2.1.2. Phenolic Acid-Gene Interactions in T2DM Pathogenesis

The most common phenolic acids are caffeic acid, chlorogenic acid (the major phenolic compound present in coffee) and ferulic acid (which is esterified to hemicelluloses in cereals) [14]. 

Most of the epidemiological studies showed an inverse association of caffeinated, decaffeinated coffee and caffeine intake with T2DM in a dose-response manner (1–6 cups/day), compared with no or rare coffee consumption, supporting the hypothesis that habitual coffee consumption is associated with a substantially lower risk of T2DM [45,46]. Regarding tea intake, several meta-analyses also reported potential beneficial effects, although it is not clear whether they are in a dose-response manner [47,48,49,50]. These beneficious effects on T2DM include an association with decreased IR [50,51], but not with increased insulin secretion and postprandial effects rather than in fasting glucose metabolism [52,53].

The protective effects of coffee consumption on T2DM involve multiple mechanisms. Recent studies have suggested that chlorogenic acid produced from caffeic acid and quinic acid reduces the ability to inhibit α-amylase and α-glucosidase activities, which are key enzymes linked to T2DM [54]. In mouse preadipocyte 3T3-L1 cells, coffee reduced the accumulation of lipids during differentiation and inhibited the expression of differentiation marker genes such as *PPAR-γ*, *adiponectin*, *CCAAT-enhancer-binding protein alpha* (*C/EBP*α), *Glut 4*, and *lipoprotein lipase* (LPL) [55]. In db/db mice, chlorogenic acid has been suggested to lower the levels of fasting plasma glucose and HbA1c during late diabetes through an adiponectin receptor signalling pathway, elevating the adiponectin level in visceral fat and the adiponectin receptors in the liver and muscles. In addition, chlorogenic acid would inhibit gluconeogenesis by down-regulating hepatic glucose-6-phosphatase (G6Pase) activity, increasing glucose transport in skeletal muscles via up-regulation of AMPK and improving disordered lipid metabolism via up-regulation of hepatic PPAR-α [56].

Regarding ferulic acid, previous studies have reported an important antioxidant activity and hypoglycemic effect in different T2DM diabetic mice models. In high fat and fructose-induced Type 2 diabetic rats, ferulic acid restored normal glucose homeostasis, improving insulin sensitivity and hepatic glycogenesis and inhibiting gluconeogenesis and the expression of insulin signalling inhibitors, such as the gluconeogenic enzyme genes PEPCK and G6Pase [57]. Besides restoring blood glucose, serum insulin, glucose tolerance and insulin tolerance to a normal range, it also reduces the over-expression of hepatic *solute carrier family 2* (*facilitated glucose transporter*), *member 2 Slc2a2* (which encodes for glucose transporter GLUT 2) by impairing the binding of SREBP1c, HNF1α and HNF3β transcription factors with the *Slc2a2* promoter [58].

#### 2.1.3. Other Bioactive Compound-Gene Interactions in T2DM Pathogenesis (Polyphenols)

Numerous studies support the effect of resveratrol (trans-3,5,4′-trihydroxystilbene), found in grapes, peanuts, blueberries and red wine, in reducing diabetic complications in many organs and tissues including liver and pancreatic β-cells and in different animal models of diabetes [59]. Other effects include the improvement of glucose homeostasis, a decrease in IR, the protection of pancreatic β-cells, an improvement in insulin secretion and an amelioration of metabolic disorders [60]. Resveratrol may act as an anti-inflammatory molecule in diabetes and other chronic diseases associated with chronic activation of NF-κB, blocking the NF-κB-dependent expression of the inflammatory cytokines IL-6, IL-8, and MCP-1 [61]. In addition, resveratrol may prevent diabetes by augmenting brain-derived neurotrophic factor (BDNF), when levels are low in T2DM, and may enhance production of the anti-inflammatory lipid, lipoxin A4 [62]. Another potential mechanism may be by inducing resveratrol-mediated changes in the gut microbiome, as recent studies in obese mice suggested, showing that faecal transplantation from healthy resveratrol-fed donor improved glucose homeostasis [63].

Controversy arises when human clinical studies are considered. Although some studies have not found clear effects [64,65], many others have described the beneficial actions of resveratrol. In obese insulin-resistant adults after four weeks of resveratrol treatment, it has been observed that postprandial glucose decreased and glucose tolerance was not dose-dependent [66]. In Type 2 diabetic subjects, resveratrol supplementation significantly improved fasting blood glucose, HbA1c, total cholesterol, triglyceride, and low density lipoprotein concentrations. The intake of resveratrol also decreased IR and oxidative stress and improved insulin signalling via the AKT pathway in Type 2 diabetic subjects [67,68]. 

Curcumin in addition to resveratrol has recently been suggested as a potential bioactive product in the field of diabetic nutrition. Treatment with either curcumin (1–100 pM) or resveratrol (0.1–10 μM) was reported to enhance pancreatic β-cell function, promoting insulin secretion in mouse β-cell lines as well as in human islets. In both humans and mice, curcumin might be acting as an inhibitor of phosphodiesterases, which degrades cAMP and cGMP, and therefore improves islet insulin secretion [69]. A 9-month study of curcumin treatment in a pre-diabetic population appeared to improve overall function of β-cells, indicated by an increased HOMA-β and reduced C-peptide, and significantly prevented T2DM development in the pre-diabetic population. In addition, curcumin intervention significantly increased adiponectin levels, which is inversely correlated to the T2DM risk [70]. Previous studies have also shown an anti-inflammation activity of curcumin by downregulating other inflammatory cytokines, such as tumor necrosis factor-alpha (TNF-α), leptin and resistin, because the inhibition of the transcription factors NF-κB and Wnt/β-catenin and the activation of peroxisome proliferator-activated receptor-γ and Nrf2 cell-signalling pathways [71]. This regulation would lead to its antidiabetic effect on IR, hyperglycemia, and hyperlipidemia.

Despite the many beneficial actions of curcumin and resveratrol, controversy rises when human clinical studies are considered [64,65]. Poor solubility and absorption of resveratrol when given orally and poor curcumin stability under certain cell culture conditions emphasize the need to optimise the experimental set-up.

### 2.2. Vitamin-Gene Interactions in T2DM Pathogenesis

Vitamin D deficiency affects insulin secretion, IR, and β-cell dysfunction since it modifies the expression of numerous genes involved in immune responses, chemotaxis, cell death and pancreatic β-cell function/phenotype [72,73]. Administration of vitamin D improves impaired glucose tolerance and insulin secretion in vitamin D deficient rats by modulating the generation and effects of cytokines [74]. In addition, vitamin D enhances insulin sensitivity by stimulating the expression of insulin receptors [75] and by promoting the expression of PPAR-δ [76] (Table 2). Furthermore, vitamin D decreases the effects of systemic inflammation in T2DM and protects against β-cell cytokine-induced apoptosis by directly modulating the expression and activity of cytokines affecting IR, such as IL-1, IL-6, and TNF-α in monocytes from T2DM patients [77]. Some studies support the indirect effects of vitamin D on β-cell function via its well-recognised role in regulating extracellular calcium and calcium influx [78].

In humans, lower vitamin D levels represent a risk factor for incident T2DM [79] and hypovitamin D levels with increased parathyroid hormone (PTH) levels seems to be an independent predictor of β-cell dysfunction, IR and glycemia [80]. Patients with T2DM with established hypovitaminosis improve glycaemia and insulin secretion by Vitamin D replenishment, not only through a direct action on pancreatic β-cell function but also via regulation of plasma calcium levels, which regulate insulin synthesis and secretion [81,82]. Currently, some clinical trials have suggested the combined effects of vitamin D and calcium supplementation for an improvement in the glycemic status of vitamin D insufficient diabetic patients [83]. However, a study selection trial that compared vitamin D3 supplementation with placebo or a non-vitamin D supplement in adults with normal glucose tolerance, prediabetes, or T2DM showed no effect of vitamin D3 supplementation on glucose homeostasis or diabetes prevention. The moderate degree of heterogeneity among subjects and the short-term follow-up duration of this trial could make the potential effects of vitamin D3 supplementation in diabetic patients inconclusive [84,85]. 

Vitamin A is essential for pancreas development, islet formation and function [86]. However, there is controversy about the effectiveness of vitamin A, retinol and its carrier proteins, retinol binding protein (RBP) and transthyretin for treating diabetes since the effects on insulin secretion seem to be dependent on its metabolites. Retinoic acid is a potent inductor of pancreatic differentiation [87]. Vitamin A (all-trans-retinol) is required for both maintenance of pancreatic β-cell and α-cell mass and for glucose-stimulated insulin secretion in adult mice [88], although studies on the use of supplements in T2DM humans have shown inconclusive results [89]. RBP might affect glucose homeostasis through the activity of GLUT. This is supported by the observation that, enhanced serum RBP4 levels appear to be a trigger for the development of systemic IR both in animal models and in humans [90,91]. Moreover, the antioxidant properties of carotenoids (pro-vitamin A compounds) may also protect against T2DM [92], although previous studies found little evidence for an association between dietary intake of lycopene, a powerful antioxidant carotenoid compound, and the risk of T2DM in women [93].

Regarding the antidiabetic effects of Vitamin E and tocopherols, although results from animal studies suggested an association between Vitamin E consumption and a lower risk of T2DM [94], several randomised interventional clinical trials not only failed to confirm that there was a beneficial effect in preventing or treating T2DM but suggested an aggravation of some diabetes affections [95,96,97].

Biotin is also required for the normal expression of critical carbohydrate metabolism genes and for glucose homeostasis, such as *forkhead box A2* (*Foxa2*), the *pancreatic and duodenal homeobox factor transcription factor* (*Pdx1*), *hepatocyte nuclear factor 4alpha* (*Hnf4α*), *insulin* (*Ins*), *glucokinase* (*Gck*), *Calcium Voltage-Gated Channel Subunit Alpha1 D* (*Cacna1d*) and *acetyl-CoA carboxylase* [98] (Table 2). In the Type 2 diabetic rat model induced by HFD and low-dose of STZ, biotin supplementation exerted antioxidant, anti-hyperlipidaemic, anti-inflammatory and anti-hyperglycaemic effects, increasing the level of insulin, probably through modulation of PPAR-γ, IRS-1 and NF-κB proteins [99].

The role of ascorbic acid in diabetes treatment remains unclear since it can also be a pro-oxidant under certain in vitro conditions and it can glycate proteins despite its beneficial preventive antioxidant properties. In vitro studies suggested that appropriate concentrations of vitamin C in pancreatic β-cells may have beneficial effects since the number of α- and β-cells increased after vitamin C supplementation [100]. Different studies have found lower levels of ascorbic acid in the plasma of diabetic patients and experimental diabetic animal models [101,102] and a strong inverse association between plasma vitamin C levels and T2DM risk [103]. Administration of a high dose of vitamin C inhibited insulin secretion in rat islets in a dose-dependent manner [104].

Clinical trials of vitamin C supplementation in diabetic populations have shown controversial results. In some Type 2 diabetic patient studies, administration of vitamin C increased plasma vitamin C levels and whole body glucose disposal thus improving both fasting blood glucose and HbA1c levels [105,106]. However, in others, administration of oral vitamin C was unable to completely replenish the plasma level of vitamin C and did not improve endothelial dysfunction or IR [107] or even suggested that supplements of vitamin C may actually increase the risk of cardiovascular disease mortality [108].

Despite their antioxidant properties and effects in the reduction of diabetic complications in diabetic mice models [109], supplementation of B vitamins in general has not been proposed for the primary prevention of T2DM. In fact, recent studies have suggested that long-term exposure to high levels of the B vitamins niacin, thiamin and riboflavin may correlate with prevalence of obesity and diabetes. In fact, niacin may induce glucose intolerance, IR and liver injury [110]. Furthermore, it has been observed that riboflavin in non-toxic doses prevents cytokines-induced p38 phosphorylation and IL-6 upregulation on insulinoma NIT-1 cells and isolated rodent islets [111]. Nicotinamide, known to promote differentiation of mouse embryonic stem cells into insulin producing cells [112,113,114], also induced insulin gene expression by increasing *v-maf avian musculoaponeurotic fibrosarcoma oncogene homolog A* (*MafA*) expression in INS1-1 β-cells [115] (Table 2).

### 2.3. Amino Acid-Gene Interactions in T2DM Pathogenesis

Dietary protein and amino acids also modulate insulin secretion and/or contribute to the maintenance of β-cell function [116,117]. The mechanisms function by influencing gene and protein expression in pancreatic islets and activating the PI3K/PKB/mTOR pathway, which results in improved insulin release [118,119]. In this respect, it has been suggested that dietary components of germinated brown rice improves glycemic control by downregulating the gluconeogenic genes *Fbp1* and *Pck1* in Type 2 diabetic rats [120]. In general, the mechanisms proposed for the amino acid effects on glucose homeostasis and blood glucose control included a direct insulinotropic effect on insulin secretion from the β-cell, the role of precursors for liver glucose synthesis, and a potential effect on insulin signalling [121]. Recent studies have suggested that supplementation with casein hydrolysate would attenuate the NLRP3-ASC inflammasome activity, a molecular platform mediating the processing of IL-1β in response to infection and stress conditions, improving insulin sensitivity and glucose tolerance in vitro and in mice fed with HFD [122].

Overall, the role of dietary protein on glucose control through endogenous production of glucose varies depending on the experimental conditions, the protein and carbohydrate content of the meal and the window of metabolic investigation after the meal. In T2DM patients, protein ingestion augmented postprandial insulin release and led to the attenuation of postprandial elevation in circulating glucose concentration [123,124]. Regarding the effects of animal protein intake on T2DM risk, several prospective cohort studies have found associations between animal protein intake and T2DM risk highly dependent on the source and type of animal protein and presence of others food components. Epidemiological studies indicate that higher intake of low-fat and fat-free fermented dairy foods are associated with lower T2DM risk [125,126,127] but more epidemiological evidence is necessary to elucidate the real association among intake of protein supplements and T2DM risk.

Amino acids are potentiators of glucose-induced insulin release [117] promoting slow cytosolic calcium oscillations in mouse pancreatic islets [116]. Four amino acids, namely arginine, leucine, isoleucine and valine are largely responsible for the observed effects. Branched-chain amino acids (BCAAs) isoleucine, leucine and valine are important as nutrient signals and metabolic regulators [128]. Epidemiological studies have shown that BCAAs plasma concentrations and metabolism are altered in T2DM. In obese and T2DM animal models, low circulating levels of adiponectin impair the BCAAs catabolism, which leads to accumulation of these amino acids through a down-regulation of mitochondrial phosphatase 2C (PP2Cm) expression mediated by the AMPK signalling pathway [129].

Increased plasma BCAAs have been associated with IR and levels of HbA1c. Accumulation of BCAAs has been suggested to promote mitochondrial dysfunction, linked to stress kinase stimulation and β-cell apoptosis which are frequently related to IR and T2DM. Furthermore, elevated circulating BCAAs levels have been found to be a reliable prediction of TDM2 especially in Caucasians and Hispanics and not only in presymptomatic individuals, but also in those classified as prediabetic or diagnosed as Type 2 diabetic [130,131,132]. Despite the numerous studies, it is not clear yet whether increased BCAAs levels are simply markers of IR or whether they are direct contributors to IR and loss of action [133,134,135,136].

Among BCAAs, l-leucine is unique in inhibiting NO synthesis from l-arginine in endothelial cells, activating glutamine: fructose-6-phosphate aminotransferase (GFAT) [137] and may modulate cardiovascular homeostasis in IR activating the mechanistic target of rapamycin (serine/threonine kinase) (mTOR) complex, a key regulator in cell growth and proliferation [138,139,140]. Leucine also appeared to enhance pancreatic β-cell insulin secretion by its ability to allosterically activate glutamate dehydrogenase, which has also been reported for other amino acids, such as phenylalanine [141].

Despite numerous studies having suggested a causal role of high levels of BCAAs in impairing insulin signalling or IR [142,143], other observations indicate that BCAAs activation of the mammalian target of rapamycin complex 1 (mTORC1), which generates an inhibitory feedback loop on insulin receptor substrate proteins, is not necessary or sufficient to trigger IR [144]. Recent work using untargeted metabolomics in a T2DM rat model consistently found that BCAA levels were not elevated until six months post-onset of diabetes, supporting the idea that the increase in BCAA level is not enough to elicit IR and T2DM in that model [145].

Elevated circulating levels of the aromatic amino acids phenylalanine and tyrosine have also been linked to the development of T2DM, acting as indirect markers of insulin sensitivity, not only in T2DM patients but also in presymptomatic individuals [130,131,146,147,148]. On the other hand, in those clinical trials, glycine was found to be reduced, possibly due to an increase in the gluconeogenesis or glutathione consumption by increased oxidative stress [130,146,147,149,150].

The insulinotropic effect of arginine in the presence of glucose could be explained by directly depolarising the plasma membrane of β-cells, activating the Ca^2+^ channels resulting in the influx of Ca^2+^ which triggers insulin exocytosis [116,151].

Arginyl-fructose, which is widely distributed in red ginseng, has antidiabetic effects attributed to it by suppressing carbohydrate absorption in the gastrointestinal tract [152]. Arginyl-fructose has potential as a pharmacological agent for glycemic control since it significantly reduces postprandial blood glucose levels in rodents and humans. In contrast, glucose-related biomarkers including HbA1, insulin, and C-peptide levels were not significantly improved by dietary supplementation with arginyl-fructose [153,154]. 

### 2.4. Dietary Fat-Gene Interactions and Their Role in T2DM

Although numerous qualitative analyses on the intake of different types of fat have found inconsistent associations between intake and T2DM risk [155,156], consumption of a high fat diet (HFD), mainly in saturated fatty acids (SFAs), is believed to be associated with an increased risk of T2DM. In addition, a correlation between a higher intake of SFAs and decreased insulin sensitivity has been demonstrated in several cross-sectional studies [125,157,158,159]. In animal models, an increase in plasma fatty acid concentration is associated with an increase in intracellular fatty acyl-CoA and diacylglycerol concentrations. As a consequence, insulin activation of IRS-1 and IRS-1-associated PI3-kinase activity is inhibited, resulting in a decrease in insulin-stimulated glucose transport activity [160]. In addition, T2DM C57BL/6J mice fed with HFD upregulated proteins involved in pancreatic β-cell proliferation and downregulation of glutathione peroxidase gene (*Gpx1*) implicated in the antioxidant defenses of β-cells and in the regulation of *MafA* expression, which is important in the regulation of insulin expression [161]. Reports supporting the potential unhealthy effects of palm oil due to the high palmitic acid content in the development of T2DM have suggested that chronic exposure of pancreatic islets to palm oil inhibited glucose-induced expression of prepro-insulin, as well as *Pdx1* and *MafA* expression, causing β-cell failure [162,163]. The mechanism of downregulation of insulin gene expression by palmitate involves the inhibition of PDX1 nuclear translocation, and therefore blocking *MafA* gene expression [164]. Besides preproinsulin mRNA expression, palmitate also reduces *Slc2a2* and *Gck* probably through the inhibition of *Pdx1* mRNA [165]. Elevated levels of fatty acids lipotoxicity have been described to be mediated by de novo synthesis of ceramide which can be formed from palmitate but not from oleate, explaining the fact that both palmitate and oleate inhibit insulin secretion but only palmitate impairs insulin gene expression [166,167]. In addition, saturated fatty acid palmitate but not unsaturated oleate, induces the activation of the NLRP3-ASC inflammasome causing caspase-1, IL-1β and IL-18 production leading to reduced glucose tolerance and insulin sensitivity [168]. On the other hand, monounsaturated fatty acid-enriched HFD improves insulin sensitivity in mice, attenuates adipose IL-1β secretion and maintains adipose AMPK activation when compared to SFAs-HFD–fed mice [169]. Moreover, we recently reported that an extra virgin olive oil intervention ameliorate non-alcoholic steatohepatosis was induced by a high fat “Western-style” diet in mice [170]. Overall, mice and in vitro experiments suggest that high levels of palmitate cause an alteration to genes involved in lipid metabolism, inflammation and oxidative stress [171]. The possible mechanism of SFAs to induce inflammation and IR in adipocytes, which would imply involvement of oxidative stress through ROS in a toll-like receptor-4 and 2 (Tlr4, Tlr2), mediated inflammatory signalling. Furthermore, activation of c-jun N-terminal kinase (JNK) also appeared to be essential to Tlr2-, as well as Tlr4-induced IR and oxidative stress [172,173,174].

Intervention studies in humans where SFAs intake was reduced via replacement with unsaturated fatty acids showed contradictory data that was observed in animal models: whilst some favored both liver and visceral fat accumulation with a detrimental role in T2DM development and progression [175], others showed improved insulin sensitivity but no effect on insulin secretion [176] whilst others showed no significant effects [177]. Recently, the EPIC-InterAct case-cohort study in people with incident T2DM reported that different SFAs have different effects on T2DM incidence [178]. Specifically, myristic, palmitic, and stearic acids were all positively associated with incident T2DM while pentadecanoic acid, heptadecanoic acid and arachidic acid were inversely associated with incident T2DM. Lauric, myristic, and palmitic acids can raise total and LDL cholesterol, increase coagulation, induce IR, and promote inflammation.

Regarding monounsaturated fatty acids (MUFAs), oleic acid has been suggested to improve glucose control and insulin sensitivity [179]. The effects observed when replacing saturated fatty acids with oleic acid may be due to partial removal of the SFAs biological effect, any constituent of the diet, or, to the combination of foods/nutrients rather than to oleic acid. However, some studies indicate oleic acid has some effects on transcription factors involved in lipid homeostasis, such as SREBPs [180]. Similarly, palmitoleic acid also increases the transcription activity of SREBP1c, apart from enhancing the binding of SREBP1c to FAS promoter and decreasing the phosphorylation of NF-κB, p65 and the expression of proinflammatory cytokines [181]. In vitro approaches indicated that *cis-*palmitoleic acid can influence pancreatic β-cell survival, insulin secretion, skeletal muscle insulin response and adipocyte metabolism, On the other hand, prospective studies have shown a positive association between higher circulating palmitoleic acid and improved insulin sensitivity or lower incident type T2DM [182,183].

T2DM is associated with increased blood concentrations of inflammatory biomarkers, including C-reactive protein (CRP), which leads to a low-grade inflammation that is the mechanism underlying IR. Epidemiological, human intervention, animal and cell culture studies have supported a beneficial role for dietary *n*-3 polyunsaturated fatty acids (PUFAs) in low-grade chronic inflammation situations, among others, by reducing CRP [184,185]. In overweight/obese patients with impaired fasting glucose or impaired glucose tolerance, *n*-3 PUFAs reduced glycemia and fasting plasma insulin [186], while in T2DM patients with omega-3 supplementation, a high ratio of eicosapentaenoic acid/docosahexaenoic acid (EPA/DHA) contributed to a greater decreasing tendency in plasma insulin and HbAc1, although with no statistically significant results [187]. The anti-inflammatory and preventive inflammation-driven disease effect of *n*-3 PUFAs is related to NLRP3 inflammasome activation and subsequent inhibition of caspase-1 activation and IL-1β secretion [188]. In addition, EPA has been suggested to repress Srebp-1c, which may lead to a lower lipid accumulation in pancreatic cells and an enhancement of insulin secretory mechanism [189,190].

Linoleic acid is the most prevalent omega-6 PUFAs in the human diet, found in some studies to increase insulin sensitivity [191]. Although it has been shown to activate NF-κB [192], dietary intakes were not associated with CRP, IL-6, soluble TNF receptor (sTNFR) 1, or sTNFR2 concentrations in humans [193], therefore having only a limited effect on inflammation [194].

## 3. Diet-Gene Interaction and Risk of T2DM

For a decade, genome-wide association studies (GWAS) on T2DM have been conducted in a variety of populations of different ancestries and more than 200 T2DM-related genetic variants have been identified by GWAS so far [5,195]. Not all, but many of the gene variants found are located in genes related to the insulin secretion pathway, insulin signalling, pancreatic β-cell dysfunction, and IR pathway [196]. However, gene variants have been described in genes that apparently would not be candidates for greater susceptibility to T2DM. In addition to the genetic architecture of T2DM susceptibility, environmental factors are suggested to play a key role in the etiology of T2DM. Dietary factors in particular may interact with genetic variants to modulate the risk of T2DM [197].

All this makes it very complex to establish T2DM risk values according to allelic variants, due to: (i) the existence of genetic variants that increase T2DM risk independently of the type of diet (ii) genetic variants related to diet that modify some glucose metabolism systems, such as fasting glucose levels and IR, but do not modify the risk of T2DM (iii) genetic variants that present a greater risk of T2DM but this risk is modified depending on the type of diet (iv) genetic polymorphisms that modify risk according to other parameters such as ethnic class and obesity. All this makes it somewhat difficult to clearly establish cause-effect and therefore, the association between a gene variant and the risk of developing T2DM.

### 3.1. Gene Variants Associated with Insulin Stimulus-Secretion Coupling and T2DM

Among the gene variants strongly associated with the risk of developing T2DM in various human populations are several genes involved in the WNT-mediated signalling pathway, which induces the expression of genes involved in pancreas development and in glucose homeostasis, such as incretin-expressing genes that potentiate insulin secretion. One of the most important is the *TCF7L2* gene encoding the transcription factor TCF7-like 2 located on chromosome 10 [198]. In several studies in white European [199], Indian [200], Japanese [201], Mexican American [202], Chinese [203] and African populations [204], *TCF7L2* showed a strong association with the odds of developing T2DM being increased by 30–50%. Several genetic polymorphisms have been described in the *TCF7L2* gene associated with an increased risk of T2DM (rs12255372 G<T, rs7903146 C>T), which interact with the diet modifying susceptibility to T2DM. In this sense, it is important to differentiate between the improvement in parameters related to glucose metabolism and the risk of T2DM. The Malmö Diet and Cancer Study (MDCS) cohort conducted by Hindy et al. 2016 showed that high fibre intake improved fasting plasma glucose and insulin levels, an improvement in IR values (HOMA-IR) and lower levels of glycosylated haemoglobin. However, the T2DM risk in individuals carrying the risk allele *TCF7L2* rs12255372 or *TCF7L2* rs7903146 was higher when fibre consumption was high. In contrast, individuals who did not carry the risk allele showed a decrease in T2DM risk with fibre intake [205].

No interaction was observed between polymorphisms and consumption of carbohydrates, fats or proteins and the incidence of T2DM [206] in MDCS cohort.

On the other hand, an EPIC sub cohort study (EPIC-InterAct study) was carried out and an interaction was noted between *TCF7L2* variant rs12255372 and coffee intake, with an inverse association between coffee consumption and T2DM among carriers of the diabetes risk allele (T) [207].

In MDCS cohort other allelic variants of genes encoding transcription factors involved in the WNT signalling pathway with greater susceptibility to T2DM that is modified by the diet are *ZBED3* rs4457053 G>A and *NOTCH2* rs10923931 G>T in which fibre consumption protects against the susceptibility to T2DM with higher fibre consumption in the risk allele carriers (T) for the case of *NOTCH2* and in individuals homozygous for the risk factor (GG) in the case of *ZBED3* [205].

The protein encoded by the gene solute carrier family 30 (zinc transporter) member 8 (*SLC30A8*) is a zinc efflux transporter protein necessary for the accumulation of zinc in intracellular vesicles. The expression of this gene is very high in pancreas, especially in pancreatic islets. Zinc is required for insulin biosynthesis and the maturation of insulin secretory granules [208]. Genetic polymorphisms have been described in this gene that increase T2DM risk. Thus, the polymorphism rs13266634 C>T was associated with lowered β-cell function and a 14% increase in diabetes abundance per risk (C) allele [209]. A significant interaction between rs13266634 and serum levels of trans-β-carotene and γ-tocopherol was identified. Higher levels of trans-β-catotene nutrient factor appeared to have a protective effect in individuals with the risk allele. In contrast, high levels of γ-tocopherol produced adverse effects in individuals with the risk allele [210]. Additionally, in a 14-cohort meta-analysis study, an interaction was observed between the allelic variant *SLC30A8* rs11558471 A>G, conferring higher levels of fasting plasma glucose to A allele carriers, and zinc consumption. In a 14-cohort meta-analysis study, total zinc intake had a stronger inverse association with fasting glucose levels in individuals carrying the glucose-raising A allele of rs11558471 *SLC30A8*, compared with individuals carrying the G allele [211].

There exists a common genetic variant in the glucose-dependent insulinotropic polypeptide receptor gene (*GIPR* rs10423928 T>A) which is related with a reduced insulin release and an increase in T2DM risk. In the Swedish population-based Malmö Diet and Cancer cohort, it has been studied the relationship between the mentioned genetic variant, macronutrients and fiber intake, body mass index (BMI) and T2DM risk. It has been observed that when AA-genotype people follow high-fat, low-carbohydrate diets, the T2DM risk decreases. On the other hand, two thirds of the people homozygous for the T-allele resulted benefitted when they follow high-carbohydrate, low-fat diets [212].

A meta-analysis of 14 cohort studies for the interactions between the glucokinase regulatory protein (*GCKR*) variant rs780094 G>A and whole grain intake demonstrated a possible relationship between the gene variant and whole grain intake. In fact, people with the insulin-raising allele rs780094 that followed a larger whole grain intake had a smaller reduction in fasting insulin compared to subjects without the variant [213].

The *S100A9* gene is located in region 1q21, a high susceptibility region to T2DM, and codes for the calcium binding protein S100A9 (calgranulin B). The variant *S100A9* (rs3014866 C>T) was associated with protection against T2DM development in several populations, such as individuals carrying the less frequent allele (T) who had a lower T2DM risk than those who did not carry the allele (CC homozygous individuals). In the intervention trial study in 3 diverse populations (CORDIOPREV: Coronary diet Intervention with Olive Oil and Cardiovascular prevention; GOLDN: Genetics of Lipids Lowering Drugs and Diet network and BPRHS: Boston Puerto Rican health Study) related to the interaction of polymorphism with diet, individuals with the non-protective variant (CC) had a higher HOMA-IR value than individuals carrying the allele (T) when the association between intake of saturated fatty acids (SFAs) versus carbohydrates was high, and that this difference disappeared when intake of SFAs/carbohydrates was low [214]. This may be one of the reasons why individuals with the CC polymorphism are at increased risk of T2DM and that diet may reduce this risk.

The transient receptor potential vanilloid 1 (*TRPV1*) gene is involved in energy and glucose metabolism. TRPV1 activation increases insulin sensitivity and potentiates glucose-stimulated insulin secretion. A Korean Genome Epidemiology Study demonstrated that individuals with the minor alleles of the *TRPV1* single nucleotide polymorphisms (SNPs) rs161364 T>C and rs8065080 T>C were negatively associated with the prevalence of T2DM. They also determined that carriers of the minor allele of both SNPs have a lower risk of diabetes with a high-fat diet but individuals with the major alleles are at a higher risk of T2DM when consuming high-fat diets [215].

In a small case-control study examining interactions between magnesium intake and loci in *TRPM6* (transient receptor potential cation channel, subfamily M, member 6) associated with T2DM, in older women carried out over 10 years, it reported that women who were carriers of 2 rare alleles from nonsynonymous SNPs in *TRPM6* (rs3750425 and rs2274924) had nearly 5 times the odds of T2DM when their magnesium intake was less than 250 mg/d [216].

The SNP rs3786897 G>A in the peptidase D (*PEPD*) gene has been associated with the risk of T2DM in Asian individuals [217]. PEPD protein plays an important role in the recycling of collagen metabolisms, while it is shown to have a profound impact on β-cell function [218]. In a case-control study examining the genetic effect of the *PEPD* rs3786897 A allele on the risk of T2DM may be abolished when n-3 PUFA intake is high [219] (Table 3).

### 3.2. Gene Variants Related to IR and T2DM

Peroxisome proliferator-activated receptor-γ (*PPAR-γ*) is a transcription factor that directly regulates target genes, mediating lipid metabolism and adipocyte differentiation. The genetic variant Pro12Ala rs180282 in *PPAR-γ* has been associated with the T2DM risk and BMI [232]. The less frequent *PPAR-γ* Ala12 variant reduces the risk of T2DM and is positively associated with insulin sensitivity. DESIR (Data from an Epidemiological study on the Insulin Resistance Syndrome) cohort study indicates high fat intake increases T2DM risk in Pro homozygous individuals [220].

Adiponectin (encoded by *ADIPOQ*) is an important adipocytokine that is secreted by adipocytes and plays a key role in the inflammatory response that is associated with insulin-resistant states and T2DM. In recent years, the association between SNPs of the *ADIPOQ* gene and T2DM has been reported in the Asian population in particular [233]. Interactions of variants of the adiponectin gene with carbohydrate intake [29] and fat intake [30] have been explored. Significant dose-response interactions were identified between the *ADIPOQ* rs1501299 G>T polymorphism and the dietary intake of carbohydrates in a prospective study of 673 patients with T2DM. This previous study demonstrated a connection between the gene-nutrient interactions of rs1501299 G>T polymorphism and the level of carbohydrate intake modulating fasting plasma blood glucose and glycosylated hemoglobin [221]. Other gene-nutrient interactions include rs2241766 T>G and a diet high in *n*-3 PUFAs. The MARINA trial study demonstrated that individuals who had a high intake of *n*-3 PUFAs showed a decreased risk of T2DM [222].

The *IRS1* gene encodes insulin receptor substrate 1 (IRS1), a protein central to insulin signalling pathways. Two genetic variants (rs7578326 and rs2943641) near *IRS1* were identified by GWAS to be associated with T2DM [223,234]. *IRS1* variants rs7578326 G allele and rs2943641 T allele were associated with a lower risk of IR and lower fasting insulin in two independent populations of different ancestries (GOLDN cohort study and the BPRHS cohort study). These associations appeared to be modulated by dietary factors, especially the dietary SFAs-to-carbohydrate ratio, MUFAs, and carbohydrate quantity and quality. Thus, low consumption of MUFAs, low total fat consumption and a low SFAs to carbohydrate ratio decrease resistance to insulin and plasma insulin, but not when consumption is high [223]. Another longitudinal cohort study also explored the relationship between *IRS1* rs2943641 and circulating levels of 25-hydroxyvitamin D 25(OH)D. Puerto Rican adult females homozygous for the minor allele rs2943641T with higher circulating 25(OH)D showed a lower risk of IR and T2DM compared to carriers of the major allele (C) [224].

The *CAV2* gene encode the major protein components of caveolae. These are invaginations of the plasma membrane. These proteins are important for several cellular functions as signal transduction, lipid metabolism, cellular proliferation, apoptosis, differentiation and trafficking. The case-control study of the European prospective Investigation into Cancer and Nutrition-Post dam cohort demonstrated a significant relationship between the *CAV2* rs2270188 G>T polymorphism and fat and SFAs intake with respect to T2DM. Homozygous individuals of the rare T-allele showed a 100% greater risk of T2DM when daily fat intake was increased from 30% to 40% in energy value, and an increase in dietary SFAs from 10% to 20% energy value predicted approximately 200% greater risk of T2DM. However, homozygotes for the G allele and heterozygotes do not have increased risk of T2DM with higher fat or SFAs consumption [225] (Table 3).

### 3.3. Other Genetic Variants and T2DM

The fat mass and obesity-associated gene (*FTO*) is located on chromosome 16 and contains nine exons. Actually, it has been described several SNPs of this gene that have been regularly associated with obesity risk. Nevertheless, there isn’t a consistent association between obesity risk alleles and T2DM. A meta-analysis of 62 case-control studies from different regions (including Asia, Europe and North America) demonstrated that the rs9939609 T>A and rs8050136 C>A SNPs contributed to an increased risk of T2DM [235]. A case-control study, with 3430 T2DM patients and 3622 healthy people without difference in their BMI, showed steady gene-diet interactions with adherence to the Mediterranean diet for individuals carrying the *FTO*-rs9939609 variant. Moreover, in the presence of a low Mediterranean diet adherence, people of the variant alleles showed a higher T2Dm risk. On the contrary, with a high Mediterranean diet adherence, these associations were not observed [226].

Dysregulation and genetic variations in the Circadian Locomotor Output Cycles Kaput genes (*CLOCK*), which are responsible for the circadian system, have been associated with T2DM [236]. A longitudinal study, undertaken with 7098 participants in the PREDIMED trial analysed the association between the SNP *CLOCK* rs4580704 C>G and the incidence of T2DM. G-allele carriers presented lower T2DM risk than CC homozygous subjects. The protective association of the G-allele against T2DM incidence was stronger and of higher statistical significance in the Mediterranean diet (High MUFAs) intervention group than in the control group (Low MUFAs) [227]. The interaction between the cryptochrome 1 (*CRY1*) rs2287161 G>C variant and several diabetes-related traits (fasting glucose and insulin, HOMA-IR and QUICKI index) has been studied in The Mediterranean and North American Cohort. The authors showed that a significant increase in HOMA-IR index and fasting insulin, as well as a decrease in QUICKI was associated with a higher carbohydrate intake in people homozygous for the minor C allele [228].

The melanocortin-4 receptor (*MC4R*) gene is a receptor for the α-melanocyte stimulating hormone (α-MSH) which regulates food intake and energy homeostasis. In a systematic review study a consistent interaction between adherence to a Mediterranean diet and the *MC4R* rs17782313 T>C was demonstrated. When adherence to the Mediterranean diet was low, carriers of the variant allele for *MC4R* rs17782313 (C) had a higher risk of T2DM than homozygous participants for the major allele (T). When adherence to the Mediterranean diet was high, however, there was no association between this polymorphism and T2DM [226,229].

The *Perilipin* gene (*PLIN*) regulates adipose metabolism and has been associated with several risk factors for diabetes, including obesity, weight gain and IR, in both American [237] and Chinese women [238]. Although an interaction between the allelic variants and an increased risk of T2DM has not been found, the analysis of the interaction between these variants and diet showed a significant gene-diet interaction between the *PLIN* 11482 G>A and the *PLIN* 14995 A>T polymorphisms with dietary fat and carbohydrate intake. A cross-sectional study of Asian women with these SNPs showed women homozygous for the minor allele A are at increased insulin resistance when SFA intake is high and carbohydrate intake is low. Notably, these gene-fat interactions were observed only for SFAs, but not for MUFAs or PUFAs [230].

Intestinal fatty acid-binding protein 2 (*FABP2*) Ala54Thr polymorphism (rs1799883) is associated with risk of T2DM. Carriers of the Thr54 allele are more susceptible to T2DM than those which are homozygous for Ala54 [231]. Fat intake and insulin sensitivity have also been studied [239] and it was shown that insulin sensitivity improved in subjects with the Thr54 allele of the *FABP2* polymorphism when SFAs were replaced by MUFAs and carbohydrates in 11 case-controlled meta-analysis studies (Table 3).

## 4. The Role of Epigenetics in the Onset of T2DM

As previously mentioned, T2DM occurs as a consequence of the interaction between genetic and environmental factors. One of the most important environmental factors to consider is diet. GWAS have identified about 100 loci that influence the risk of developing T2DM. Most of them are responsible for alterations in the development of pancreatic islets and/or their function and only a few act through insulin signalling [240]. However, these variants have a reduced effect on the risk of T2DM and are only able to explain at most 10–15% of T2DM inheritance [4]. Thus, if T2DM is a disease in which the hereditary component is important and the genetic variants can explain only a minimal part of that inheritance, this means epigenetic modifications could explain the relationship between environmental factors and the risk of developing T2DM. The proposed explanation is that epigenetic changes occurring throughout life caused by environmental factors (such as diet) can modify gene expression and affect susceptibility to T2DM. In this regard, one of the major changes that has occurred in the Western world and that undoubtedly contributes to the increase in the prevalence of T2DM is an increased caloric intake and reduced energy expenditure, the latter caused by a sedentary lifestyle.

Epigenetics is a form of genomic control that produces changes in the structure of chromatin, without generating changes in the DNA sequence. These changes in chromatin originated by external stimuli are capable of activating or silencing gene expression. There are three types of epigenetic mechanisms: (i) DNA methylation; (ii) post-transcriptional modification of histones; (iii) Mechanisms based on non-coding RNA (microRNA & long non-coding RNA). The first modifies the accessibility to the genes by inducing transcriptional repression. The second includes acetylation, methylation, phosphorylation, ubiquitination and SUMOylation of certain histone amino acids. These processes induce chromatin condensation and consequently, the phenomena of gene activation and/or silencing. Finally, non-coding RNAs can regulate gene expression by acting on the protein synthesis machinery at the translational and post-transcriptional levels. Of special note is the role of microRNAs (miRNAs) and long non-coding RNAs (LncRNAs). Numerous studies have shown that epigenetic modifications are able to induce IR and damage β-cells, thereby causing insufficient insulin release and T2DM [241].

It has been established in experimental studies that patients with T2DM have reduced β-cell mass [242]. In addition, this is known to occur due to external factors, sometimes of a nutritional nature and may make it difficult to maintain the state of differentiation of β-cells. The control of β-cell mass during development and in adulthood is regulated by master genes that act as a balance to regulate β- and α-cell mass. In this regard, paired box gene 4 (*Pax 4*) is necessary for the existence of mature β-cells and Arista-less-related gene homeobox (*Arx*) for the formation of α-cells [243]. Thus, in an experimental study with pancreatic islets from patients with T2DM, the *PAX4* gene is hypermethylated and therefore silenced [244]. In contrast, in rodents, the *Arx* gene is methylated and repressed in β-cells [245]. Variations in DNA methylation also affect insulin release processes. For example, the incretin hormone glucagon-like peptide-1 (GLP-1) increases insulin release acting through its receptor (GLP1R). It has been confirmed that in isolated pancreatic islets of patients with T2DM, hypermethylation of the *GLP1R* gene occurs, thus reducing its expression [246].

Post-transcriptional modifications of histones are also able to regulate β-cell mass. It has been shown that in animals lacking histone deacetylase 5 and 9 expression, β-cell mass is increased. Conversely, in mice, with increased deacetylase 4 and 5 expression, there is decreased β-cell mass [247]. Moreover, it is known that although β-cell mass is established before adulthood, it can be modified according to metabolic demands, such as obesity and pregnancy. Indeed, failures in this compensatory capacity can lead to the onset of diabetes [248]. Interestingly, epigenetic modifications can regulate this adaptive proliferation response. This would be done, as it has been demonstrated in mice, by modifying the expression of the cyclin dependent kinase inhibitor 2A gene (*Cdkn2a*) by post-transcriptional modifications of histones [249]. Another fundamental master gene for the maintenance of the β-cell phenotype, and for its correct functioning, is *Pdx1*. It has been shown in mice that the association between this gene and the histone deacetylases varies according to the levels of circulating glucose [250]. Histones also play a role in insulin secretion in response to glucose. For example, histone methyltransferase Set7/9 promotes the expression of important genes such as v-maf musculoaponeurotic fibrosarcoma oncogene family, *MafA* and *Slc2a2* for insulin secretion. In fact, a depletion of Set7/9 in mouse pancreatic islets induces a decrease in insulin release in response to glucose [251].

miRNAs regulate processes related to insulin signalling as well as inflammatory processes. Both phenomena are directly involved in the onset of IR. The role of miRNAs in the development of IR and the onset of T2DM has been studied in different metabolic tissues (liver, adipose, muscle and pancreas) both in humans and mice. In addition, it has been suggested that the microvesicular transport of miRNAs could be a mechanism by which miRNAs act as local and systemic mediators of IR [252]. Studies indicate that the miRNA 143–145 cluster intervenes in IR produced by obesity. Mice that do not express this cluster are protected against IR induced by obesity caused by a high caloric intake. In contrast, conditioned overexpression of miRNA worsens obesity-induced IR [253]. The let-7 family of miRNAs also participates in the regulation of IR. Mice fed a HFD and overexpression of let-7 had impaired glucose tolerance and IR, despite having normal insulin production and secretion levels [254]. Moreover, the administration of anti-miRNA let-7 partially diminished the effects of HFD on IR in experimental studies on mice and humans [255]. The miRNA 200 family also participates in the regulation of insulin sensitivity. Specifically, inhibition of miRNA 200a in the hypothalamus of ob/ob mice increases leptin receptor expression, decreasing body weight and increasing insulin sensitivity [256]. miRNA 223 knockout mice exhibit higher IR when fed an HFD. In addition, they present a higher degree of inflammatory stress [257]. It has been known for several years that the miRNA29 family negatively regulates the insulin signalling pathway in adipocytes [258]. In addition, increased miRNA29 expression in the livers of diet-induced obese mice and Zucker diabetic rats has been demonstrated. Most interestingly, miRNA29 levels were normalised after treatment with pioglitazone, in both models [259]. miRNA 375 is particularly important for blood glucose regulation. It is expressed at high levels in β-cells, contributes to the maintenance of the β-cell phenotype and enhances insulin synthesis in IR mouse models. In this respect, ob/ob mice show increased miRNA 375 expression. Conversely, mice lacking miRNA 375 have low insulin synthesis and hyperglycemia [260,261]. In addition, experimental studies have shown that miRNA 375 is abundantly expressed during human pancreatic islet differentiation [262].

Aside from dietary modifications, certain nutrients are capable of affecting miRNA expression. It is known that chronic hyperglycemia and hyperlipidemia are capable of modifying gene expression and inducing β-cell damage or dysfunction leading, ultimately, to apoptosis. This process is known as glucotoxicity and lipotoxicity. Excess glucose and fatty acids lead to stress on the rough endoplasmic reticulum of the β-cells, which in turn causes an increase in the production of reactive oxygen species (ROS) [263]. In other tissues it has been shown that ROS can induce modifications in DNA methylation [264], which could also occur in β-cells. Elevated levels of glycosylated hemoglobin indicating a chronic blood glucose elevation, have been found to correlate positively with increases in methylation of the promoter regions of the *INS* and *PDX1* genes in human pancreatic islets [265,266]. Regarding miRNAs, it has been shown in rat pancreatic islets that prolonged exposure of β-cells to SFAs, such as palmitate, increases the expression levels of miRNA 34a and miRNA 146a [267]. Also, glucose is able to modify the expression of miRNA 9, 30d, 124a, 130a, 132, 133, 212 and 335 [268].

### 4.1. Epigenetic Modifications as Biomarkers of T2DM

Epigenetic modifications can be used as epigenetic biomarkers to detect recent β-cell death. In experimental studies, in mice and humans, it has been shown that the insulin promoter has low methylation levels in endocrine cells, but its methylation level is higher in the rest of the cells in the body [269]. Dying cells release genomic DNA into the bloodstream. When a high proportion of lowly methylated versus highly methylated insulin promoter is found, this is an indicator of recent β-cell death [3]. In the GOLDN Cohort, it was observed that the methylation levels of the ATP binding cassette gene subfamily G member 1 (*ABCG1*, important for cholesterol transport) were significantly associated with fasting insulin levels and IR values in patients with T2DM [270]. In an observational study in the Twins-UK Cohort, it was noted that methylation changes occurred in mucosa-associated lymphoid tissue lymphoma translocation gene 1 (*MALT1*) and G-protein receptor 6 (*GPR6*) gene which indicated T2DM progression [271]. Different degrees of DNA methylation in the adipose tissue of patients with T2DM compared to healthy individuals has been found in peroxisome proliferator activated receptor gamma (*PPAR-γ*), insulin receptor substrate 1 (*IRS1*) and transcription factor 7-like 2 (*TCF7L2*) genes [272]. Finally, decreased methylation of the septin 9 (*SEPT9*) gene has been identified in an experimental study using pancreatic islets of patients with T2DM [273].

The presence of miRNAs have also been proposed as potential biomarkers for different diseases including T2DM. This idea is supported by several facts: (i) They are present in human fluids (e.g., plasma, serum, urine, saliva and tears); and (ii) They are very stable at room temperature and show good resistance to freeze-thaw changes. In T2DM there is the additional advantage that compared to other traditional biomarkers (blood, glucose and insulin levels), certain miRNAs have a greater postprandial stability [252]. Nonetheless, the use of miRNAs as biomarkers of T2DM poses three problems. The first is that it is very difficult to determine the precise origin of most circulating miRNAs. Because pancreatic islets make up a tiny fraction of our body’s cells, the amount of plasma miRNAs that come from the pancreatic islets is probably negligible. Secondly, the expression of a specific miRNA is affected by numerous physiological and pathological conditions and, accordingly, it varies. Finally, it is important to have information that relates the plasma miRNAs to SNPs associated with T2DM. Studies are beginning to appear that lead us to believe that miRNAs could be used as biomarkers of T2DM. It has been shown that members of the let/7 family of miRNAs are able to modulate insulin sensitivity and glucose metabolism. A nutritional intervention study found that in healthy young women, reducing the dietary glycemic load increased plasma levels of let-7b by up to eight times [274]. In addition, plasma values of let-7a and 7f are significantly lower in patients with T2DM. In a case-control study, it was found that in treated patients, the levels were normalised when compared to those of the control subjects [275].

### 4.2. Fetal Developmental Memory

It is known that a large portion of epigenetic footprints are established during the fetal development period. Thus, an adverse intrauterine environment can be “memorized” by generating specific methylation patterns. These patterns may increase the risk of developing T2DM in adulthood, something akin to “metabolic reprogramming”. Within the impact of maternal nutrition, it is important to consider the lipid profile, circulating blood glucose, overweight and obesity, and malnutrition. The proposed explanation is that transient nutritional changes occurring throughout the prenatal period, and possibly affecting the future risk of developing T2DM, are produced as a consequence of epigenetic changes. This hypothesis is based on three assumptions: (i) Epigenetic modifications can occur in response to environmental factors, including food; (ii) Epigenetic changes can be maintained stable throughout life; (iii) These changes can be transmitted to the next generation through gametes.

In animal models, the effects of different nutritional models on the development of T2DM in the F1 and F2 generations have been studied in young female rodents in gestation and during lactation. The nutritional models usually employed are low protein diets, caloric malnutrition and HFD, although other animal models include intrauterine growth retardation (IUGR). Thompson et al. (2010) found that the pancreatic islets of rats subjected to IUGR have an alteration in gene expression, together with different methylation patterns at approximately 1400 CpG sites [276]. In this same IUGR model, a permanent reduction in *Pdx1* expression is produced in β-cells due to epigenetic changes occurring during development. This reduction in *Pdx1* expression has been correlated with the onset of T2DM [277]. On the other hand, Sandovici et al. [278] found that when low protein diets were fed to rats during pregnancy and lactation, there was an increase in methylation of *Hnf4α* gene promoter, as well as a reduction in the active marks of histone H3 (H3K3me) acetylation in pancreatic islets. Furthermore, the glucose intolerance produced in mice subjected to a caloric malnutrition model is transmitted to the F1 generations and not to the F2 generations [279]. This is due to the appearance of different regions that are methylated and are resistant to epigenetic erasure during the early stages of embryogenesis [280]. In HFD models maintained during pregnancy, an increased risk of obesity and T2DM has also been observed. When female mice were fed an HFD from the fourth week prior to pregnancy until the end of lactation, male mice born in the first generation developed obesity and IR when they reached adulthood [281]. Another widely used model to study fetal environment effects on the development of T2DM includes the induction of gestational diabetes in pregnant females in order to generate a hyperglycemic environment in the uterus. In this case, the offspring of these mothers had a defect in insulin secretion leading to glucose intolerance. In addition, the male offspring of the females born in an environment of intrauterine hyperglycemia also developed the same phenotype [282]. In both cases, the defective insulin secretion was due to hypermethylation of the DNA methylation region, together with a concomitant reduction in the expression of insulin growth factor-2 and H19.

Although there is less information available, there is one study that identifies the existence of a possible “metabolic reprogramming” of paternal origin. Ng et al. crossed male rats subjected to a HFD with control female rats and found that their offspring had altered β-cell function as well as a differential expression of 642 genes in their pancreatic islets [283]. Moreover, the study by Dunn et al. found that the second generation of male offspring also developed the same alterations [284]. The existence of transmission of a metabolic phenotype mediated by dietary modifications up to the second generation and through the paternal line suggests that epigenetic mechanisms are contributing to this process. To verify whether this generational transmission of phenotypes is mediated by epigenetic factors linked to the germ line, Dunn et al. analysed the F3 generation phenotypes. It was found that the F3 generation females, who were born to the F2 generation males, also showed the same phenotype [281]. Another study also indicates that the induction of HFD-mediated obesity in male mice causes glucose intolerance in their offspring [285]. Finally, in models where prediabetes was induced (with HFD and low-dose of STZ) in male mice, their offspring developed IR and glucose intolerance. When studying gene expression in the islets, a differential expression of 402 genes in the islets of the offspring was found. In addition, when genome-wide DNA methylation analysis was performed there were more than 800 regions showing different methylation patterns in the islets of the offspring. Most interestingly, this study demonstrated that prediabetes in the fathers altered methylation patterns in their sperm. Finally, a significant portion of the hypermethylated and hypomethylated intragenic regions overlapped in the sperm and pancreatic islet cells.

In humans, the most solid data comes from the analysis of what occurred during the famine in Holland between 1944 and 1945. There was a period of approximately five months during which there was a significant food restriction that affected pregnant women in different periods of their gestation. In addition, these women had adequate food before and after this short period. The study and follow-up of the children, after reaching adulthood, who were affected by this famine during their intrauterine life provides much information on how feeding in the prenatal period may subsequently affect the risk of developing T2DM.

The cohort study of Ravelli et al. showed that adults who were born near this period of famine had lower glucose tolerance. The authors of this study concluded that poor intrauterine nutrition could cause permanent changes in the glucose homeostasis process and that these changes were much more evident when those individuals developed obesity [286]. Subsequently, De Rooij et al. found, in the Dutch Famine Birth Cohort, that this glucose intolerance was mediated by a defect in insulin secretion [287]. Moreover, it has been proven, in the cohort study of Hillier et al. that the impact of such alterations can even be transmitted to the next generation [288]. In a recent study in a cohort of people exposed to famine in Bangladesh during their intrauterine developmental stage, their increased predisposition to T2DM was shown to be due to epigenetic changes (mainly changes in DNA methylation). These epigenetic changes vary according to the intrauterine development time frame during which famine was experienced [289]. A similar conclusion was reached by Heijmans et al., in the Dutch Hunger Winter Families Study [290]. In addition, these authors found that the effects of famine on epigenetic modifications were more critical if famine occurred during the embryonic developmental period.

Furthermore, studies in the Dutch Famine Birth Cohort found that when the fetuses were subjected to insufficient feeding during any period of their gestation, there was an increase in the LDL:HDL ratio upon reaching adulthood [291]. This increases the risk of developing T2DM, as it is well established that an increase in LDL levels predisposes to T2DM.

In contrast, a population-based cohort study with women who develop obesity during pregnancy, have IR. The excess nutrients consumed by the mother reach the fetus which in turn releases its own insulin, thus increasing the fetal uptake of glucose and fats causing these infants to be born with a higher weight [292]. When these children reach adulthood, they are exposed to an increased risk of developing obesity and T2DM. Studies by Wellen et al. indicate that in cell culture lines, the ATP-citrate lyase enzyme is essential for histone acetylation [293]. This enzyme is critical in the conversion of citrate through glycolysis into acetyl-CoA. Curiously, the acetylation of histones is more evident in periods when there are sufficient glucose inputs. There is another historical cohort from Northern Sweden (Överkalix cohort) that demonstrates that overfeeding in children may increase the risk of T2D in their grandchildren [294]. Finally, as occurs with male rats, a historical cohort study based on the Dutch Famine Population showed that the children of men who experienced prenatal malnutrition are more obese and therefore at greater risk of developing T2DM [295].

## 5. Conclusions

T2DM is a multifactorial disease which arises from complicated interactions between the genetic makeup and environmental factors. Among these factors, many studies (experimental, case-control, cohort and nutritional interventions) have shown the impact of diet on the development of T2DM. Several cohort studies have also shown that diet can generate epigenetic modifications throughout the lifetime that affect gene expression and consequently, susceptibility to T2DM. Moreover, these epigenetic modifications might play a relevant role in the inheritance of T2DM.

Nutrition is a very important factor that modulates expression of genes involved in metabolic pathways related to T2DM pathogenesis. As previously discussed, different macro and micronutrients, as well as food bioactive compounds positively affect the expression of genes involved in insulin synthesis, stimulus-secretion coupling, protection of β-cells against gluco-lipotoxicity, inflammation and oxidative stress, and IR. However, this jigsaw is not so easy to solve. Nutrigenetics give us the shape of the pieces, but we still need the drawing of the pieces of the puzzle and the resulting picture we want to reproduce. 

The information about the pieces is provided by nutrigenomics. Linkage analysis, candidate gene approaches, genome-wide association studies and sequencing have provided us with plenty of information that allows a better understanding of the genetic landscape of T2DM. This disease has a strong genetic predisposition and the discovery of the existence of genetic variants associated with T2DM and their related traits has shed light on the different individual risks. There are cohort and nutritional intervention studies showing that people with higher genetic predisposition should avoid particular dietetic patterns that are more harmful in the variation of a specific T2DM-related phenotype.

**Figure 1:**
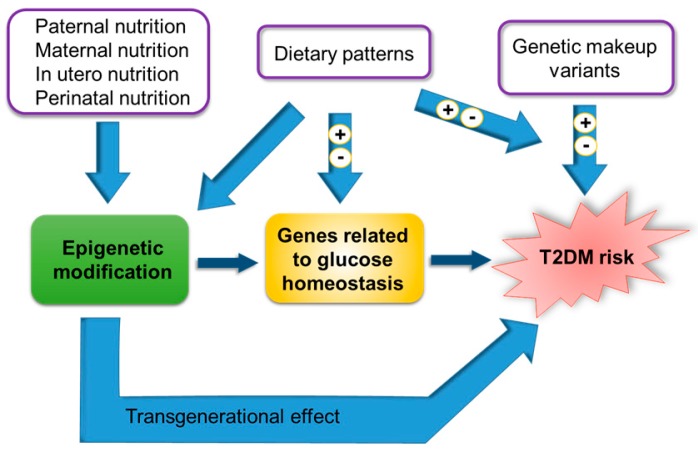
Schematic diagram of the role of nutritional, genetic and epigenetic factors in the development of T2DM. Nutrients, dietary patterns and genetic makeup (SNPs) have a direct impact on T2DM risk. Parental nutrition, prenatal and perinatal nutrition induce epigenetic modifications that increase the susceptibility of T2DM development during adulthood. The epigenetic modifications can be inherited to following generations. +: Increase; −: decrease.

The picture to be reproduced in our puzzle appears when the epigenetic factors come into the scene. Nutrigenetics and nutrigenomics are not able to completely explain the complex relationship between environmental factors and genetic susceptibility to T2DM. In fact, the overall risk explained by genetic variations is small. Epigenetic could be the missing link that gives shape to the puzzle. The rational is that nutrients and dietetic patterns experienced during one’s lifetime, including the prenatal and neonatal periods, induce epigenetic modifications that affect gene expression and change the individual disease risk present in our genetic makeup (Figure 1). In addition, these epigenetic modifications could be behind the possible transgenerational inheritance of T2DM.

In this review, we have tried to provide clues in order to demonstrate that the relationship between diet and genes in T2DM is not easy to understand, nor is it easy to pinpoint which came first: genes or diet (or vice versa). At the moment, there are three actors: (i) Nutrient-gene interactions; (ii) Individual genetic variants; (iii) Epigenetic modifications. Considering these three actors, we could likely write the script and it should not take long, because considering the global pandemic of T2DM and the broad spectrum of T2DM patients, the movie is currently being shown without the screenwriters. This approach will result in patient centered, more accurate precision-medicine. 

## Figures and Tables

**Table 1:** Flavonoids-gene interactions in the development of type 2 diabetes (T2DM).

Flavonoids	Genes and Gene Products Affected	Function	Experimental Model	Reference
**Flavan-3-ols**				
catechins	↑ *PI3K*, ↑ eNOS signalling system	↓ Hyperglycemia	STZ-diabetic rats	[20]
epigallocatechingallate (EGCG)	↑ *Irs2*, ↑ *Akt*, ↑ *Foxo1*, ↑ *Pdx1*	↑ Viability of β-cell, ↑ Insulin secretion	RIN-m5F cells	[21]
	↑ *AMPK* ↑ *PI3K/Akt* pathway	↓ IR	dexamethasone-induced rat L6 cells	[22]
	↑ *PI3K* phosphorylation	↑ Glucose uptake	differentiated rat L6 myotubes	[23]
	↑ *Bcl-2*	↓ Apoptosis, ↑ Glucose uptake	RINm5F cells	[24]
	↓ *l-cpt-1*, ↓ *Ddit3*, ↓ *Ppp1r15a*, ↓ *Cdkn1a*	↑ Insulin secretion, preserve islet structure	db/db mice	[25]
	↑ Glucokinase, ↑ Acyl CoA oxidase-1 (ACO-1) ↑ Carnitine palmitoyl transferase-1 (CPT-1) ↓ Phosphofructokinase ↓ *Glut 1*	↑ Insulin secretion ↓ Gluconeogenesis ↑ Glycolysis	db/db mice and ZDF rats	[26]
**Flavanones**				
naringin or hesperidin	↑ *Gk* (liver), ↑ *Glut 4*, ↑ PPAR-γ	↓ Hyperglycemia	C57BL/KsJ-db/db mice	[30]
naringin	↑ PPAR-γ, ↑ *Hsp*	↓ Hyperglycemia, ↓ Hyperinsulinemia, ↓ IR, ↑ β-cell function	HFD-STZ-induced T2D rats	[31]
	↓ PEPCK ↓ G6Pase	↓ Hyperglycemia, ↓ Hyperinsulinemia, ↓ IR, ↑ β-cell function	HFD-STZ-induced T2D rats	[30]
**Flavonols**				
quercetin	↑ Antioxidant enzyme activity ↓ Nitric oxide (NO) production	↓ Lipid peroxidation, ↑ β-cell preservation	STZ-induced DM mice	[32]
	↑ *Nrf-2/HO-1* activation ↓ NF-κB	↓ High glucose induced apoptosis	Dorsal root ganglion neurons of rats	[33]
	↓ *Cdkn1a*	↓ Hyperglycemia, ↑ Insulin plasma levels ↑ Pancreatic cell proliferation	STZ-induced DM mice	[35]
	↓ interferon-γ, ↓ IL-1α, ↓ IL-4	↓ Circulating markers of inflammation	C57Bl/6j mice	[36]
	↑ adiponectin, ↓ NOx levels in plasma, ↓ TNF-α	↓ Dyslipidemia ↓ Hypertension ↓Hyperinsulinemia	obese Zucker rats	[37]
**Flavones**				
luteolin, apigenin	↓ iNos ↓ NF-κB	↓ Apoptosis ↓ IL-1β- and IFN-γ-mediated inhibition of insulin secretion	RINm5F cells	[38]
**Isoflavones**				
daidzein and genistein	↓ PPAR-γ ↓ *Glut-2* ↓ SREBP-1	↑ Insulin secretion ↓Hyperinsulinemia	rats and pancreatic islets of rats in a HFD	[39]
genistein	↑ cAMP/PKA-dependent ERK1/2 signalling pathway	↓ Hyperglycemia ↑ Glucose tolerance, ↑ Insulin plasma levels ↑ β-cell preservation	STZ-induced DM mice	[40]
**Anthocyanins**				
	↑ heme oxygenase-1 (HO-1) gene ↑ ERK1/2 and PI3K/Akt signalling	↑ Insulin secretion ↓Apoptosis	pancreatic β INS-1 cells and primary islets	[42]
	↑ *Glut 4* ↓ retinol binding protein 4 (RBP4)	↓ Hyperglycemia ↑ Insulin sensitivity	T2D KK-A(y) mice	[43]
	↑ *Glut 4* ↑ AMPK, ↑ PPAR-α, ↓ Acetyl-CoA carboxylase ↑ Acyl-CoA oxidase (ACO), ↑ Carnitine palmitoyltransferase-1A-(CPT-1)	↓ Hyperglycemia ↑ Insulin sensitivity	T2D KK-A(y) mice	[44]

**Table 2:** Vitamin-gene interactions in the development of T2DM.

Vitamins	Genes and Gene Products Affected	Function	Experimental Model	References
**vitamin D**				
	↑ Human insulin receptor gene	↑ Insulin sensitivity	U-937 human promonocytic cells	[75]
	↓ TNF-α, IL-6, IL-1, and IL-8	↓ β-cell cytokine-induced apoptosis	CD14^+^ monocytes isolated from peripheral blood insulin-treated T2DM patients	[77]
**Biotin**				
	↑ *Foxa2*, ↑ *Pdx-1* ↑ *Hnf-4α* ↑ Ins, ↑ Gk, ↑ *Cacna1d*, ↑ *Acac*	↑ Insulin secretion ↑ Islet function	BALB/cAnN Hsd mice	[98]
	↑ PPAR-γ ↑ IRS-1 ↓ NF-κB	↑ Antioxidant ↓ Hyperlipidaemia ↓ Inflammation ↓ Hyperglycaemia	T2DM rat model induced by high-fat diet (HFD) and low-dose STZ	[99]
**Riboflavin**				
	↓ IL-6 upregulation	↓ Cytokines-induced p38 phosphorylation	Insulinoma NIT-1 cells and isolated rodent islets	[111]
**Nicotinamide**				
	↑ *MafA*	↑ Insulin synthesis	INS-1 cells, pancreatic islets	[115]
	↑ *Pdx-1*	Pancreatic β-cell differentiation	Mouse embryonic stem cells	[112,113,114]

**Table 3:** List of gene-nutrient interactions in the development of T2DM.

**Genes Related with Stimulus-Secretion Coupling**					
**Gene**	**SNP**	**T2D Risk**	**Types of Studies/Dietary Factors**	**Effect of Interaction between SNP and Dietary Factors**	**References**
*TCF7L2*	rs12255372 G>T	Allele-T risk increase	MDCS cohort/fibre intake	Higher risk in individuals carrying the allele-T. Smaller risk in individuals homozygous GG	[205]
			EPIC InterAct study/Coffee Intake	Smaller risk in individuals carrying the allele-T	[207]
	rs7903146 C>T	Allele-T risk increase	MDCS cohort/fibre intake	Higher risk in individuals carrying the allele-T. Smaller risk Individuals homozygous CC	[205]
*ZBED3*	rs4457053 G>A	Allele-G risk increase	MDCS cohort/fibre intake	Smaller risk in individuals homozygous for the risk factor (GG)	[205]
*NOTCH2*	rs10923931 G>T	Allele-T risk increase	MDCS cohort/fibre intake	Smaller risk in individuals carrying the allele-T	[205]
*SLC30A8*	rs13266634 C>T	Allele-C risk increase	NHANES cohort study/*trans*-β-carotene intake	Smaller risk in individuals carrying the allele-C	[209,210]
			NHANES cohort study/γ-tocoferol intake	Higher risk in individuals carrying the allele-C	[209,210]
	rs11558471 A>G	Allele-A increases levels of fasting plasma glucose	14-Cohort meta-analysis study/zinc intake	Smaller levels of fasting plasma glucose in individuals carrying the allele-A	[211]
*GIPR*	rs10423928 T>A	Allele-A risk increase	MDCS cohort/fats and carbohydrates intake	Smaller risk in individuals homozygous for de risk factor AA consuming high-fat, low-carbohydrate diets. High-carbohydrate, low-fat diets benefit of the population homozygous for the T-allele	[212]
*GCKR*	rs780094 C>T	Allele-T risk increase	14-cohort meta-analysis study/whole-grain intake	Smaller risk in individuals carrying the allele-T	[213]
*S100A9*	rs3014866 C>T	Allele-T risk decrease	Intervention trial in CORDIOPERV, GOLDN and BPRHS/SFAs:carbohydrates ration	Ration high: Individuals with the non-protective variant (CC) had a higher HOMA-IR. Ration low: Individuals with the non-protective variant (CC) had a normal HOMA-IR.	[214]
*TRPV1*	rs161364 T>C	Allele-C risk decrease	Cohort study/high fat diet intake	Smaller risk in individuals carrying the allele-C Higher risk in individuals carrying the allele-T	[215]
	rs8065080 T>C				
*TRPM6*	rs2274924 C>T	Allele-T risk increase	Cases-control study/magnesium intake <250 mg/day	Higher risk in women carrying the allele-T	[216]
	rs3750425 C>T				
*PEPD*	rs3786897 G>A	Allele-A risk increase	Cases-control study/High *n*-3 PUFAs	The risk disappears in individuals carrying the allele-A	[219]
**Genes Related with Insulin Signaling**					
**Gene**	**SNP**	**T2D**	**Dietary Factors**	**Effect of Interaction between SNP and Dietary Factors**	**References**
*PPAR-γ*	rs180282 C>G	Allele-G risk increase	DESIR cohort study/high fat intake increases	Higher risk in individuals carrying the allele-T	[220]
*ADIPOQ*	rs1501299 G>T	Allele-T risk increase	Cohort study/carbohydrate intake	Higher fasting blood glucose and HbA1C concentrations in individuals carrying allele-T	[221]
	rs2241766 T>G	Allele-G risk increase	MARINA trial study/hight *n*-3 PUFAs	Smaller risk in individuals carrying the allele-G	[222]
*IRS1*	rs7578326 A>G	Allele-G lower risk of IR and lower fasting insulin	GOLDN and BPRHS cohort studies/low total fat and SFA:carbohydrate ratio intake	Decrease resistance to insulin and plasma insulin	[223]
	rs2943641 C>T	Allele-T lower risk of IR and lower fasting insulin	GOLDN and BPRHS cohort studies/low fat and SFA:carbohydrate ratio intake	Decrease resistance to insulin and plasma insulin	[223]
			Cohort study/vitamin D intake	Smaller risk in individuals carrying the allele-T	[224]
*CAV2*	rs2270188 G>T	Allele-T risk increase	Cases-cohort study/high fat or SFA intake	Higher risk in individuals carrying the allele-T.	[225]
**Other Genes**					
**Gene**	**SNP**	**T2D**	**Dietary Factors**	**Effect of Interaction between SNP and Dietary Factors**	**References**
*FTO*	rs9939609 T>A	Allele-A risk increase	Cases-control study/adherence to Mediterranean diet	The risk disappears in individuals carrying the allele-A	[226]
	rs8050136 C>A	Allele-A risk increase	Cases-control study/adherence to Mediterranean diet	The risk disappears in individuals carrying the allele-A	[226]
*CLOCK*	rs4580704 C>G	Allele-G risk decrease	PREDIMED trial study/high MUFA intake	Lower risk in individuals carrying the allele-G	[227]
*CRY1*	rs2287161 G>C	Allele-C risk increase	Cohorts Mediterranean and North American study/high carbohydrate intake	Increase in HOMA-IR index, fasting insulin and a decrease in QUICKI	[228]
*MC4R*	rs17782313 T>C	Allele-C risk increase	Systematic review study/adherence to Mediterranean diet	The risk disappears in individuals carrying the allele-C	[226,229]
*PLIN*	rs11482 G>A	Allele-A risk increase	cross-sectional study/SFAs:carbohydrates ration	Ration high: women homozygous for AA increased HOMA-IR	[230]
*FABP2*	rs1799883 G>A	Allele-A risk increase	11-cases-control meta-analysis study/MUFA intake	Decrease in HOMA-IR index	[231]

## Abbreviations

T2DM	Type 2 diabetes Mellitus
STZ	Streptozotocin
PI3K	Endothelial phosphatidylinositol 3-kinase
EGCG	Epigallocatechin gallate
Ins	Insulin
IR	Insulin resistance
IRS	Insulin receptor substrate
AMPK	AMP-activated protein kinase
GLUT	Glucose transporter
Ppp1r15a	Protein phosphatase 1, regulatory subunit 15A
Cdkn1a	Cyclin-dependent kinase inhibitor 1A
ACO-1	Acyl CoA oxidase-1
CPT-1	Carnitine palmitoyl transferase-1
HbA1c	Glycated hemoglobin
PPAR	Peroxisome proliferator-activated receptor
PEPCK	Phosphoenolpyruvate carboxykinase
HFD	High-fat diet
HSP	Heat shock proteins
NO	Nitric oxide
NF-κB	Nuclear factor kappa B
CRP	C-reactive protein
IFN-γ	Interferon-gamma
IL	Interleukin
G6Pase	Glucose-6-phosphatase
RBP	Retinol binding protein
Pdx1	Duodenal homeobox factor transcription factor
Hnf	Hepatocyte nuclear factor
Gk	Glucokinase
Cacna1d	Calcium Voltage-Gated Channel Subunit Alpha1 D
MafA	v-Maf avian musculoaponeurotic fibrosarcoma oncogene homolog A
BCAAs	Branched-chain amino acids
SFAs	Saturated fatty acids
ROS	Reactive oxygen species
MUFAs	Monounsaturated fatty acids
PUFAs	Polyunsaturated fatty acids
EPA	Eicosapentaenoic acid
DHA	Docosahexaenoic acid
GWAS	Genome-wide association studies
IRS	Insulin receptor substrate
FTO	Fat mass and obesity-associated
SNPs	Single nucleotide polymorphisms
CLOCK	Circadian Locomotor Output Cycles Kaput
MC4R	Melanocortin-4 receptor
PLIN	Perilipin
FABP	Fatty acid-binding protein
miRNAs	microRNAs
LncRNAs	Long non-coding RNAs
Pax	Paired box gene
Arx	Arista-less-related gene homeobox
GLP	Glucagon-like peptide
IUGR	Intrauterine growth retardation
LDL	Low-density lipoprotein
HDL	High-density lipoprotein
